# Possible roles of AMPK and macropinocytosis in the defense responses against Δ^9^-THC toxicity on HL-1 cardiomyocytes

**DOI:** 10.1016/j.toxrep.2021.04.014

**Published:** 2021-04-30

**Authors:** Tatsuhiko Murata, Kanako Noritake, Toshihiko Aki, Koichi Uemura

**Affiliations:** Department of Forensic Medicine, Graduate School of Medical and Dental Sciences, Tokyo Medical and Dental University, Tokyo, Japan

**Keywords:** Δ^9^-THC, ER stress, Macropinocytosis, AMPK, HL-1 cells

## Abstract

Cannabinoids are some of the most popular recreationally used illicit drugs, and are frequently consumed along with alcoholic beverages. Although the whole body effects of cannabinoids depend largely on their effects on the central nerve system, cannabinoids could harm the heart directly, due to the presence of the endocannabinoid system including cannabinoid receptor1 and 2 (CB-R1 and CB-R2) in the heart. The aim of this study is to examine the mechanism of direct cardiotoxicity of Δ^9^-tetrahydrocannabinol (Δ^9^-THC), the main psychoactive ingredient of cannabis. For this purpose, HL-1 murine atrial cardiac muscle cells were treated with 10 or 30 μM Δ^9^-THC, along with 100 mM ethanol to examine the possible synergistic effects of Δ^9^-THC and ethanol. Transcriptome analysis showed upregulation of the genes involved in the unfolded protein response (UPR), including Bip, CHOP, ATF4 and ATF6, in cells treated with Δ^9^-THC. Immunoblot analysis showed caspase3 activation, indicating apoptosis caused by ER stress in Δ^9^-THC-treated cells. Microscopic analysis showed that Δ^9^-THC enhances macropinocytosis, a process involved in the uptake of extracellular fluids including nutrients. Moreover Δ^9^-THC seemed to activate AMPK, a sensor of intracellular energy status and an activator of macropinocytosis. Finally, we found that compound C (AMPK inhibitor) aggravated cell death by Δ^9^-THC while AICAR (AMPK activator) ameliorated it. Collectively, these results indicate that the activation of AMPK is necessary for the survival of HL-1 cells against Δ^9^-THC toxicity. Macropinocytosis might serve as one of the survival pathways downstream of AMPK.

## Highlights

• Δ^9^-THC induced ER stress in HL-1 murine cardiomyocyte.
• Δ^9^-THC also induced macropinocytosis, which was mediated by AMPK.
• AMPK-macropinocytosis axis was one of the survival pathways against Δ^9^-THC.

## Graphical abstract

**Figure:**
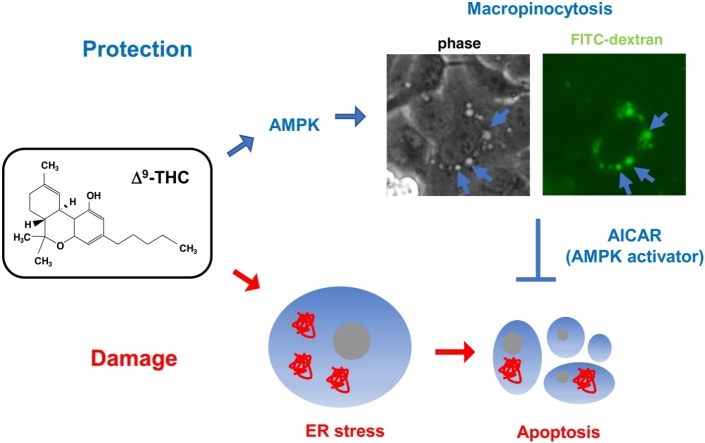

## 1 Introduction

Cannabinoids are components of the cannabis plant which includes *Cannabis Indica* and *Cannabis Sativa* as the two major subspecies. Marijuana, which is widely used as a recreational drug, is made from dried parts of cannabis plant. Although the use of marijuana is associated with relatively few deaths, adverse effects on the cardiovascular system such as arrhythmias, ischemic attack, and cardiac infarction sometimes lead to sudden cardiac death [1]. The risk of sudden death due to the cardiovascular toxicity of cannabis has recently expanded due to the worldwide distribution of synthetic cannabinoids [[2], [3], [4], [5], [6]]. Alcohol can increase the harmful effects of cannabinoids; therefore, drinking alcoholic beverages while consuming marijuana may raise the possibility of sudden cardiac death [7,8]. Although responses of the heart to cannabinoids depend highly on the individual, in general, an acute increase in heart rate is followed by decreased heart function [1]. Among naturally occurring cannabinoids, Δ^9^-tetrahydrocannabinol (Δ^9^-THC) is the most potent psychoactive constituent of marijuana [9]. Δ^9^-THC elicits its effects by binding to cell surface G-protein coupled receptors, referred to as type-1 and -2 cannabinoid receptors (CB-R1 and CB-R2), both of which are expressed in cardiomyocytes [10,11]. Although CB-R1 and CB-R2 are the main transducers of Δ^9^-THC signals, other receptors, such as G-protein coupled receptor 55 (GPR55), are also expressed in the heart and are involved in Δ^9^-THC signal transduction [12].

ER stress and subsequent unfolded protein response (UPR) are processes involved not only in cell survival but also in cell death [13,14]. Upon accumulation of unfolded proteins within the ER, an ER-resident chaperone, Bip, initiates UPR. Downstream of Bip, UPR diverges 3 ways into the IRE1α, PERK, and ATF6 pathways [13]. IRE1α can induce pro-apoptotic JNK activation [15]. The activation of PERK leads to the activation of proapoptotic CHOP transcription factor through ATF4 [14,16]. Although ATF6 induces ER-resident chaperones such as Bip and PDI for the protection of cells against ER stress, prolonged ATF6 activation results in the induction of CHOP [14]. Therefore, excessive UPR can induce apoptotic cell death through any of these 3 pathways. Caspase-12 is considered to be responsible for ER-stress-induced apoptosis in murine cells [17], though other functions, such as an inflammatory caspase, are attributed to this caspase [18]. There are many reports describing the importance of ER stress in Δ^9^-THC -induced apoptosis in various cell types [[19], [20], [21]].

Macropinocytosis is a type of clathrin-independent endocytosis in which extracellular fluids, including nutrients, antigens, and small water-soluble molecules, are taken up nonspecifically [22]. Macropinocytosis begins with protrusion of the plasma membrane through the polymerization of actin, followed by the engulfment of extracellular fluids via closure of the membrane protrusions at their distal margins. Then, the luminal space of macropinosomes is delivered to the lysosome to digest their contents. Although excessive macropinocytosis sometimes results in massive cytoplasmic vacuolization and resultant catastrophic cell death, referred to as methuosis [23], macropinocytosis ordinarily participates in cellular homeostasis, especially for tumor cell survival as a nutrients-acquiring cellular strategy [22,24]. In accordance with its role in nutrient acquisition, macropinocytosis is facilitated by AMPK [25,26], which is activated during nutrient deficiency [27]. To the best of our knowledge, macropinocytosis has not been a topic in the context of Δ^9^-THC cytotoxicity, in contrast to ER stress, which has been repeatedly reported in Δ^9^-THC-treated cells.

In this study we examined the cytotoxicity mechanism of Δ^9^-THC and/or ethanol on HL-1 murine atrial cardiomyocytes, and found ER stress as a cell death mechanism. In addition, we found AMPK activation protects the cells against Δ^9^-THC cytotoxicity. We also observed cytoplasmic vacuolization through enhanced macropinocytosis, which was activated by AMPK and therefore might be involved in the protective role of AMPK against Δ^9^-THC cytotoxicity.

## 2 Materials and methods

### 2.1 Δ^9^-THC and other reagents

Δ^9^-Tetrahydrocannabinol (Δ^9^-THC, kindly provided by Dr. Satoshi Morimoto, Kyushu University) was dissolved in DMSO (FUJIFILM Wako Pure Chemical, Osaka, Japan) at a final concentration of 100 mM and stored at −80 °C until use. Ethanol, AM251, and AM630 were purchased from FUJIFILM Wako Pure Chemical. Tauroursodeoxycholic acid (TUDCA) was from Millipore Corp (USA). 5-Aminoimidazole-4-carboxamide-1-β-d-ribofuranoside (AICAR) was from Sigma-Aldrich (St. Louis, MO, USA). Compound C was from abcam (Cambridge, MA, USA).

### 2.2 Cell culture

HL-1 mouse atrial cardiomyocyte-derived cells were kindly provided by Dr. William C. Claycomb (Louisiana State University Medical Center) [28]. The cells were maintained as recommended by the Claycomb Laboratory. In brief, cells were cultured on gelatin/fibronectin-coated dishes at 37 °C in a humidified atmosphere containing 5% CO_2_ in Claycomb medium supplemented with 10 % fetal bovine serum, 100 U/mL penicillin, 100 μg/mL streptomycin, 0.1 mM norepinephrine, and 2 mM l-glutamine. Once the cells had grown to confluency and started to beat spontaneous, the indicated concentrations of Δ^9^-THC (10 or 30 μM) and/or 100 mM ethanol were added directly to the medium. The culture dishes were sealed with laboratory film throughout the incubation period to minimize ethanol evaporation.

### 2.3 Viability assays

Cells were incubated with 30 μM Δ^9^-THC and/or 100 mM ethanol for 48 h. In some experiments, the indicated concentrations of inhibitors or activators were added to the medium 1 h before the addition of Δ^9^-THC and/or ethanol, and included throughout the incubation period. Cell viability was determined by a modified MTT assay using a Cell Counting Kit-8 (CCK-8; Dojindo, Kumamoto, Japan).

### 2.4 Western blot analysis

Cells grown on 3 cm diameter dishes were scraped, collected together with floating cells by centrifugation, and subjected to ultrasonic wave disruption in STE buffer (0.32 M sucrose, 10 mM Tris−HCl, pH 7.4, 5 mM EDTA, 50 mM NaF, 2 mM Na₃VO₄) on ice. Equal amounts of protein were subjected to SDS-PAGE, transferred to a PVDF membrane, and blocked in TBS-Tween (150 mM NaCl, 10 mM Tris−HCl, pH 7.4, 0.05 % Tween 20) containing 3% skim milk. The membranes were incubated overnight at 4℃ with specific antibodies (Supporting information, S1 Table). After washing with TBS-Tween, the membranes were incubated with peroxidase-conjugated anti-rabbit antibody (1:10,000 dilution, Promega, USA) at room temperature for 45 min. Antigens were visualized using a Western Lightning Chemiluminescence Reagent Plus Kit (Perkin Elmer Life Science, USA), and the signal intensities were quantified using Image J (ver.1.52v, National Institutes of Health, USA).

### 2.5 Microarray and quantitative real-time PCR analysis

For DNA microarray and quantitative real-time RT-PCR (qPCR) analysis, total RNA was extracted from cells using TRlzol reagent (Invitrogen, USA). For DNA microarray analysis, the total RNA was further purified using a Monarch total RNA prep kit (New England Biolabs, USA). RNA integrity was assessed by a BioAnalyzer (Agilent Technologies, USA), and DNA microarray analysis was performed using Clariom™S array (Affymetrix, Thermo Fisher Scientific, USA). DAVID Bioinformatics Resources (https://david.ncifcrf.gov/summary.jsp) were used to analyze the results. For qPCR analysis, cDNA was synthesized using oligo (dT)_15_ primer and SuperScriptⅡreverse transcriptase (Invitrogen, USA). qPCR was performed with the StepOnePlus real-time PCR system (Applied Biosystems, USA). Primers used are listed in Supporting information (S2 Table).

### 2.6 Transfection of vectors and fluorescence microscopy

Cells grown on 3 cm dishes for 24 h were transfected with plasmid vectors expressing kinectin (1–106)-GFP [29] or GFP-HSP47 [30], which were kindly provided by Dr. Erika Abe (Akita University) or Dr. Kazuhiro Nagata (Kyoto University), respectively. Vectors were mixed with Lipofectamine2000 (Invitrogen), added to the cells, and the cells were incubated overnight. After treatment with 30 μM Δ^9^-THC for 24 h, cells were observed under a fluorescence microscope (DMi8, Leica, Wetzlar, Germany).

### 2.7 Uptake of FITC-dextran

To observe macropinocytosis, we used fluid-phase tracer FITC-dextran, which is widely known as a marker for macropinocytosis [31]. Cells grown on 3 cm diameter dishes for 2 days were treated with 30 μM Δ^9^-THC and 0.5 mg/ml FITC-dextran (average M.W. 70 kDa, Sigma-Aldrich) for 24 h. The cells were then washed twice with the medium and observed under a fluorescence microscope (DMi8, Leica).

### 2.8 Statistical analysis

All data comprising more than three experimental groups were analyzed using the Tukey-Kramer statistical method for multiple comparisons. Student’s *t*-test was also used for the comparison of two experimental groups. The data are expressed as mean ± S.D. of at least three samples. P values <0.05 were considered to be statistically significant.

## 3 Results

### 3.1 Δ^9^-THC decreases cellular viabilities in HL-1 cells

**Fig. 1:**
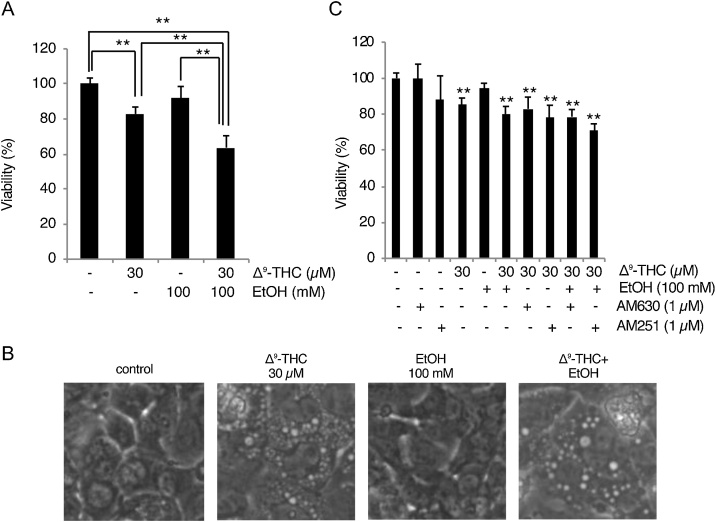
Δ^9^-THC toxicity in HL-1 cells involves cytoplasmic vacuolization. (A) Δ^9^-THC and/or ethanol decreases cell viability. HL-1 cells were treated with the indicated concentrations of Δ^9^-THC and/or ethanol for 48 h. Cellular viabilities were determined by modified MTT assay. Mean cell viability of control cells [Δ^9^-THC (-), ethanol (-)] was set to 100 %, and relative cell viabilities of Δ^9^-THC and/or ethanol treated cells are shown [mean ± S.D. (n = 6); **, p < 0.01]. (B) Cells were treated with the indicated concentrations of Δ^9^-THC and/or ethanol for 48 h and observed under light microscopy. (C) Δ^9^-THC toxicity is not ameliorated by CB-R1/2 antagonists. Cells were pretreated with 1 μM AM630 or 1 μM AM251 for 1 h and further treated with the indicated concentrations of Δ^9^-THC and/or ethanol for 48 h. Graph shows the result of modified MTT assay [mean ± S.D. (n = 6); **, p < 0.01 versus untreated group].

We first examined whether Δ^9^-THC decreases the viability of HL-1 cells, as well as whether or not ethanol affects Δ^9^-THC cytotoxicity. Given the indication that the cytotoxicity of Δ^9^-THC becomes detectable in cells at around 10 μM [32], and our previous observation that 0.5–1 % (86−172 mM) ethanol decreases the viability of HL-1 cells slightly but not significantly [33], cells were treated with 30 μM Δ^9^-THC and/or 100 mM ethanol for 48 h. The relative viabilities (mean ± S.D., expressed as % of control) of each experimental group were 82.9 ± 3.93 % (Δ^9^-THC), 92.0 ± 6.58 % (ethanol), and 63.6 ± 6.80 % (Δ^9^-THC + ethanol) (Fig. 1A). There are significant differences between Δ^9^-THC and Δ^9^-THC + ethanol groups, as well as ethanol and Δ^9^-THC + ethanol groups (Fig. 1A). Although the loss of viability in the Δ^9^-THC + ethanol group (36.4 %) was slightly higher than that of the sum (25.1 %) of Δ^9^-THC (17.1 %) and ethanol (8.0 %), there was no obvious synergistic effect between Δ^9^-THC and ethanol. Thus, the cytotoxicity of Δ^9^-THC and ethanol seems to be additive, rather than synergistic, under our experimental settings. Interestingly, we observed cytoplasmic vacuolization in Δ^9^-THC-treated cells, which was not observed in ethanol-treated cells (Fig. 1B), supporting the assumption that the toxicities of Δ^9^-THC and ethanol on HL-1 cells are independent, rather than interactive. Next, we examined whether the cytotoxic effects of Δ^9^-THC are transmitted through cannabinoid receptors. Neither AM251 (1 μM, CB-R1 antagonist) nor AM630 (1 μM, CB-R2 antagonist) could rescue the loss of viability caused by Δ^9^-THC (Fig. 1C). Thus, the Δ^9^-THC-induced loss of cell viability seems to be a CB-R1/2 -independent process.

### 3.2 Transcriptome analysis identifies ER stress- and myocardial function-related genes as the genes mostly affected by Δ^9^-THC

To obtain insight into the mechanism of Δ^9^-THC cytotoxicity, we performed transcriptome analysis. Cells were treated with 30 μM Δ^9^-THC for 24 h, and subjected to DNA microarray analysis to identify the pathways that are affected by Δ^9^-THC. Although the most significantly influenced pathway was identified as ribosome biogenesis, the expressions of genes related to ER stress and myocardial function were also identified as being increased and decreased by Δ^9^-THC, respectively (Table 1).

**Table 1:** Biological processes affected by Δ^9^-THC treatment. Upper and lower panels list biological processes that were up- and down-regulated, respectively.

Up-regulated	
Biological process	P-value
Ribosome biogenesis	1.04E-17
Response to endoplasmic reticulum stress	9.08E-07
Endoplasmic reticulum unfolded protein response	5.55E-07
Response to unfolded protein	4.51E-04
ER-associated ubiquitin-dependent protein catabolic process	8.94E-04


**Down-regulated**	
Biological process	P-value
Cardiac muscle contraction	7.00E-13
Muscle contraction	3.92E-10
Sarcomere organization	1.02E-08
Heart development	4.01E-08
Regulation of heart rate	6.65E-047
Regulation of heart rate by cardiac conduction	1.44E-06
Cardiac muscle tissue morphogenesis	1.48E-05

### 3.3 Δ^9^-THC induces ER stress and subsequent apoptosis in HL-1 cells

**Fig. 2:**
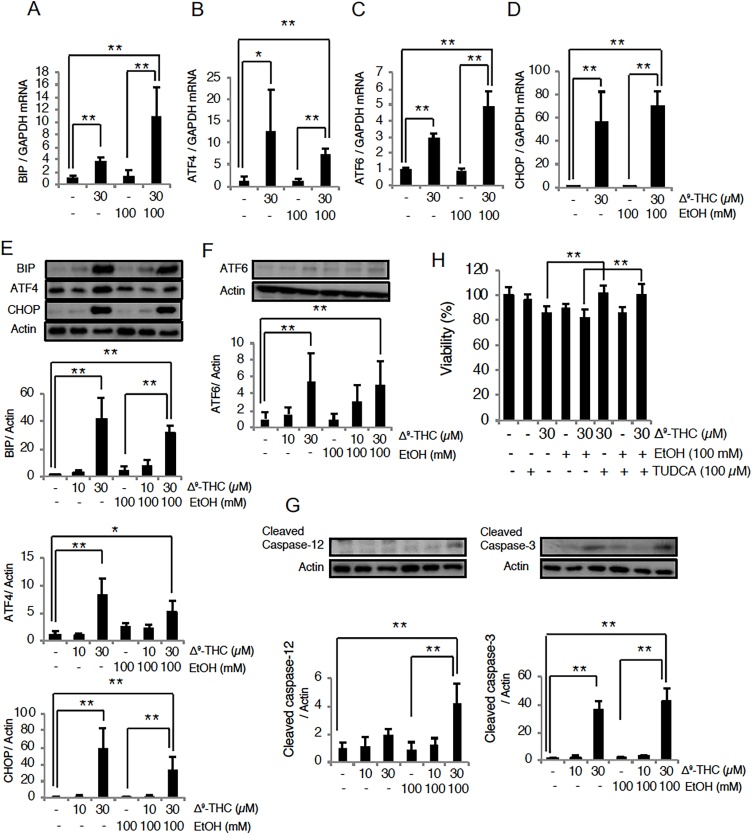
Δ^9^-THC induces ER stress responses in HL-1 cells. (A–D) Induction of ER stress genes. HL-1 cells were treated with the indicated concentrations of Δ^9^-THC and/or ethanol for 24 h. Levels of mRNAs of BIP, ATF4, ATF6 and CHOP were determined by qPCR. GAPDH was used as an internal control. Each graph shows mean ± S.D. (n = 3) **, p < 0.01; *, p < 0.05. (E–G) Increase in ER stress proteins and activation of caspases in HL-1 cells treated with the indicated concentrations of Δ^9^-THC and/or ethanol for 48 h. Levels of BIP, ATF4, ATF6 and CHOP (E and F) as well as the cleavage of caspase-12 and -3 (G) were determined by western blot analysis. Actin was also examined as a loading control. Each graph shows mean ± S.D. (n = 3) **, p < 0.01; *, p < 0.05. (H) ER stress inhibitor TUDCA alleviates Δ^9^-THC cytotoxicity. Cells were pretreated with 100 μM TUDCA, and further treated with the indicated concentrations of Δ^9^-THC and/or ethanol for 48 h. Cell viabilities were determined by modified MTT assay. Mean cell viability of control cells [Δ^9^-THC (-), ethanol (-)] was set to 100 %, and relative cell viabilities of Δ^9^-THC and/or ethanol treated cells are shown [mean ± S.D. (n = 5 or 6); **, p < 0.01].

We checked further whether ER stress responses are indeed induced by Δ^9^-THC. Cells were treated with 10 or 30 μM Δ^9^-THC with or without 100 mM ethanol for 24 and 48 h, and then evaluated by quantitative RT-PCR and western blot analysis. Both the mRNA and proteins levels of BIP, ATF4, ATF6 and CHOP, markers of the ER stress response, increased in response to 30 μM Δ^9^-THC (Fig. 2A-F), confirming the ER stress response. Although the co-administration of ethanol seemed to affect the expressions of several of the ER stress genes induced by Δ^9^-THC, there was no certain tendency for an effect of ethanol on Δ^9^-THC-induced ER stress gene expression (Fig. 2A-D). Significant increase of all the BIP, ATF4, ATF6 and CHOP gene expressions were observed in Δ^9^-THC + ethanol group compared to ethanol group (Fig. 2A-D), suggesting that these gene expressions were governed mainly by Δ^9^-THC. In contrast, only BIP and CHOP showed increased protein levels in Δ^9^-THC + ethanol group compared to ethanol group (Fig. 2, E and F). Therefore, there should be some differences between the regulations of protein and mRNA of these genes. Further, we examined the involvement of ER stress in the cytotoxicity of Δ^9^-THC using tauroursodeoxycholic acid (TUDCA), which has been shown to function as a chemical chaperone and suppresses ER stress [32,34] TUDCA pretreatment significantly alleviated Δ^9^-THC cytotoxicity (Fig. 2H). Thus, ER stress appears to be involved, at least in part, in the cytotoxicity of Δ^9^-THC on HL-1 cells. To examine whether apoptosis is involved in the observed loss of viability of Δ^9^-THC-treated cells (Fig. 1A), we evaluated the levels of the cleaved forms of caspase-12 and caspase-3. Interestingly, a significant increase in cleaved caspase-12 was observed only in the Δ^9^-THC + ethanol group, implying that Δ^9^-THC and ethanol may activate the caspase-12 pathway in a synergistic manner (Fig. 2G). In contrast, a significant increase in cleaved caspase-3 was observed in all experimental groups in which 30 μM Δ^9^-THC was included (Fig. 2G), suggesting that the loss of cell viability by Δ^9^-THC involves apoptotic cell death through caspase-3.

### 3.4 Δ^9^-THC decreases both MYH-6 and MYH-7 expression

**Fig. 3:**
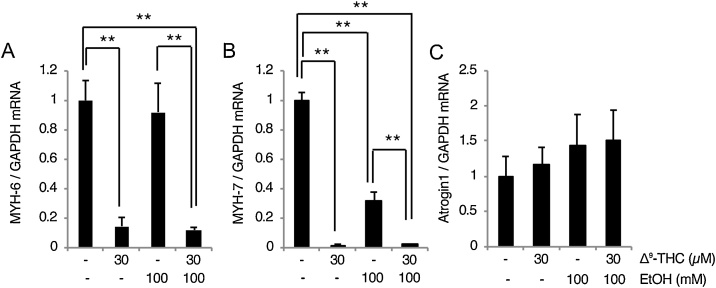
Δ^9^-THC affects myocardial function-related gene expressions HL-1 cells. HL-1 cells were treated with the indicated concentrations of Δ^9^-THC and/or ethanol for 24 h. Levels of MYH-6 and MYH-7 (A and B) as well as atrogin1 (C) mRNAs were determined by qPCR. GAPDH was also examined as an internal control. Each graph shows mean ± S.D. (n = 3) **, p < 0.01.

Given the indication of the downregulation of myocardial function-related genes (Table 1), we examined the mRNA levels of MYH-6 and MYH-7 (also known as α- and β-myosin heavy chain) and found that both are decreased by Δ^9^-THC (Fig. 3 A and B). Expression of the MYH-7 gene was also decreased by ethanol (Fig. 3 B), suggesting that ethanol by itself can affect myocardial functional gene expression. Significant decreases were observed in both MYH-6 and MYH-7 gene expression in Δ^9^-THC + ethanol group compared to ethanol group (Fig. 3 A and B), indicating that Δ^9^-THC showed decreasing effect on MYH-7 gene even under the presence of ethanol. We also found a tendency towards an increase in the expression of atrogin-1, an E3 ubiquitin ligase implicated in the degradation of myosin [35], in response to ethanol and/or Δ^9^-THC (Fig. 3C). Thus, not only the decrease in gene expression, but also protein degradation might be involved in the downregulation of myocardial functional genes.

### 3.5 Cytoplasmic vacuolization in Δ^9^-THC-treated cells involves ER dilation as well as macropinocytosis

**Fig. 4:**
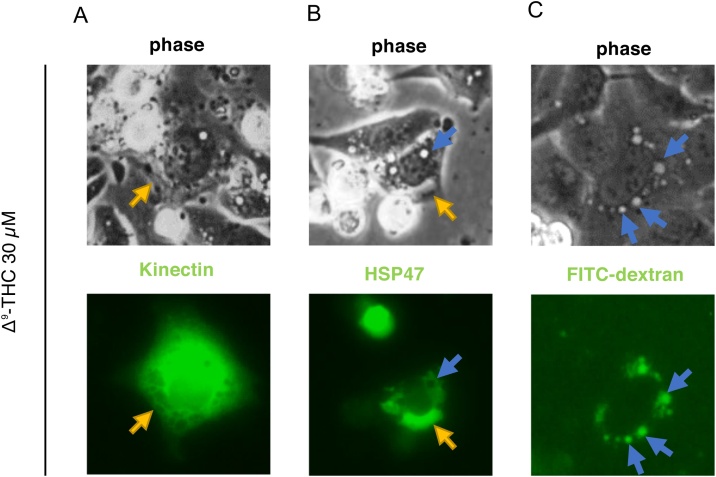
Δ^9^-THC induces cytoplasmic vacuolization through macropinocytosis in HL-1 cells. Cells were transfected with vectors kinectin (1–106)-GFP (kinectin-GFP) and GFP-HSP47, or treated with fluid phase tracer FITC-dextran, then exposed to Δ^9^-THC for 24 h. The cells were observed under a fluorescence microscope. (A) Kinectin (1–106)-GFP localized at the peripheral edge of vacuoles in Δ^9^-THC-treated cells. (B) GFP-HSP47 localized inside vacuoles in HL-1 cells. (C) Cytoplasmic vacuoles induced by Δ^9^-THC incorporated FITC-dextran. Yellow and blue arrows indicate putative ER-derived vacuoles and macropinosomes, respectively.

We next investigated the origin of the vacuoles that are observed in Δ^9^-THC-treated cells (Fig. 1B). We first suspected ER dilation as the origin of the cytoplasmic vacuoles, and so we transfected cells with plasmid vectors expressing kinectin (1–106)-GFP and GFP-HSP47, which localize to the membrane and luminal space of the ER, respectively [29,30]. In cells treated with Δ^9^-THC, kinectin-GFP localized to the edge of several vacuoles (Fig. 4A). In contrast, GFP-HSP47 localized to the lumen of large vacuoles, which were sometimes observed in Δ^9^-THC-treated cells (Fig. 4B). However, neither kinectin-GFP nor GFP-HSP47 localized to phase lucent vacuoles that have a perfect round shape (Fig. 4 A and B). We then explored the origin of these vacuoles. FITC-dextran was observed to be taken up into these phase lucent vacuoles (Fig. 4C), suggesting that they are macropinosomes. Taken together, cytoplasmic vacuolization by Δ^9^-THC involves at least two mechanisms: dilation of the ER and an enhancement of macropinocytosis.

### 3.6 AMPK is activated by Δ^9^-THC and involved in cytoprotection against Δ^9^-THC toxicity

**Fig. 5:**
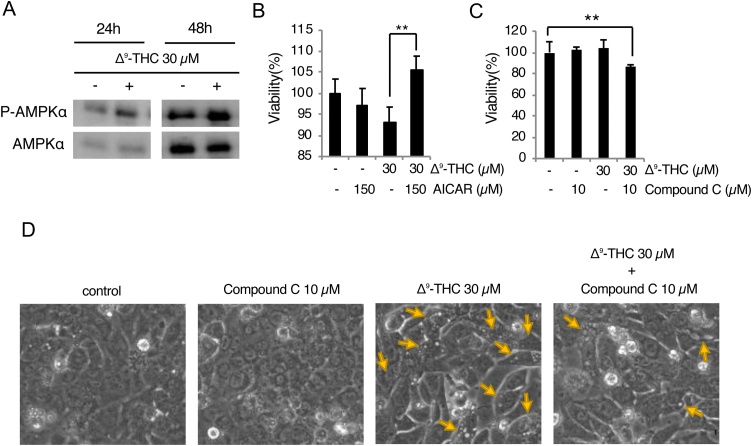
AMPK is involved in the survival of HL-1 cells during exposure to Δ^9^-THC. (A) Activation of AMPK in HL-1 cells treated with the indicated concentrations of Δ^9^-THC for 24 or 48 h. (B and C) Δ^9^-THC cytotoxicity is ameliorated by AICAR and aggravated by compound C. After pretreatment with 150 μM AICAR or 10 μM compound C for 1 h, cells were treated with the indicated concentrations of Δ^9^-THC for 24 h. Cellular viabilities were determined by modified MTT assay. Mean cell viability of control cells [Δ^9^-THC (-)] was set to 100 %, and relative cell viabilities of Δ^9^-THC treated cells are shown [mean ± S.D. (n = 5 or 6); **, p < 0.01]. (D) Δ^9^-THC-induced macropinocytosis is mitigated by compound C. Yellow arrows indicate vacuolated cells.

Given the indication of enhanced macropinocytosis (Fig. 4C) and the fact that AMPK is involved in the induction of macropinocytosis [25,26], we examined whether Δ^9^-THC activates AMPK. Levels of the phosphorylated active form of AMPK (p-AMPK) as well as total AMPK were evaluated by immunoblotting. An increase in p-AMPK levels relative to total AMPK was observed in cells treated with Δ^9^-THC for both 24 and 48 h (Fig. 5A). Furthermore, cells treated with an AMPK activator (AICAR) or inhibitor (compound C) showed significantly mitigated or aggravated Δ^9^-THC cytotoxicity, respectively, (Fig. 5 B and C). Thus, AMPK activates a survival pathway against Δ^9^-THC toxicity in HL-1 cells. In addition, pretreatment with compound C indeed mitigated cytoplasmic vacuolization in Δ^9^-THC-treated cells (Fig. 5D), confirming that AMPK is involved in the induction of macropinocytosis in these cells.

## 4 Discussion

Many studies have shown that THC induces ER stress and subsequent cell death. For example, Carracedo et al., reported that Δ^9^-THC induces ER stress and subsequent cell death though CB-R2 in several human pancreatic tumor cell lines [19]. They also reported decreased growth of pancreatic tumor cells in animals administered Δ^9^-THC, showing that Δ^9^-THC reduces tumor growth through ER stress-mediated cell death *in vivo* [19]. Lojpur et al. also reported that in human trophoblast BeWo cells, Δ^9^-THC-induced ER stress as well as apoptosis is suppressed by a mixture of CB-R1 and CB-R2 antagonists [32]. Therefore, it is a bit surprising that Δ^9^-THC-induced death of HL-1 cells could be suppressed by antagonists of neither CB-R1 nor CB-R2 (Fig. 1C). Since there are other proteins that can function as cannabinoid receptors including GPR55 [12], TRP channels [36] and PPARs [37], Δ^9^-THC might induce UPR through receptors other than CB-R1/2. Alternatively, there is a possibility that Δ^9^-THC induces ER stress and subsequent apoptosis in a manner independent of these receptors. Indeed, the possible involvement of receptor-independent processes in the action of cannabinoids has been proposed to occur in addition to receptor-mediated processes [38]. Due to its hydrophobicity, Δ^9^-THC can interact directly with membrane phospholipids, thereby altering membrane properties such as fluidity, integrity, and the barrier function of micro- as well as macro-molecules. Indeed, membrane perturbation by Δ^9^-THC has been demonstrated [39]. Thus, future investigations should be undertaken to determine whether Δ^9^-THC induces ER stress and cell death through receptors other than CB-R1, CB-R2, or whether there is a direct interaction of Δ^9^-THC with the cardiomyocyte membrane.

A massive accumulation of cytoplasmic vacuoles, most of which are derived from macropinocytosis, was observed in Δ^9^-THC-treated cells. However, the Δ^9^-THC-induced death of HL-1 cells seems not to be caused by methuosis since cell death could be suppressed by an activator of AMPK, an inducer of macropinocytosis (Fig. 5); AMPK-macropinocytosis seems to be involved in the protection against Δ^9^-THC toxicity in HL-1 cells. Macropinocytosis plays crucial roles in nutrient uptake into cells from extracellular environments [22]. The incorporation of nutrients through macropinocytosis is especially important for the survival of tumor cells as well as for normal cells encountering nutrient deficiency [22]. Indeed, a recent study demonstrated that macropinocytosis-mediated nutrient uptake plays an important role in tumor cell resistance to chemotherapy drugs [40]. Furthermore, it has been demonstrated that Δ^9^-THC inhibits glycolysis in the testis of mice as well as rats in a dose dependent manner [41]. A recent *in vitro* study has also shown that both CB-R1- and -R2-specific synthetic cannabinoids (arachidonoyl cyclopropamide and GW405833, respectively) suppress glycolysis as well as glutamine uptake in Panc1 pancreatic tumor cells [42]. Thus, it might be reasonable to suppose macropinocytosis as one of the survival mechanisms downstream of AMPK. It is worth noting that the induction of autophagy, another cellular defense mechanism against nutrient deficiency [43], by Δ^9^-THC has been repeatedly reported [21,[44], [45], [46]].

In conclusion, this is the first report showing enhanced macropinocytosis in Δ^9^-THC-treated cells. AMPK is involved not only in the induction of macropinocytosis but also in cytoprotection against Δ^9^-THC in the cells. Along with autophagy, AMPK might protect against Δ^9^-THC cytotoxicity by inducing macropinocytosis.

## Funding

This study was supported by a grant-in-aid from MEXT KAKENHI (grant number 19K19483 to K.N. and 18K19670 to T.A.).

## CRediT authorship contribution statement

**Tatsuhiko Murata:** Investigation, Validation, Data curation, Writing - original draft, Visualization. **Kanako Noritake:** Conceptualization, Data curation, Project administration, Formal analysis, Funding acquisition. **Toshihiko Aki:** Project administration, Funding acquisition, Writing - review & editing. **Koichi Uemura:** Supervision, Project administration.

## Declaration of Competing Interest

The authors declare no conflict of interest.

## References

[1] Drummer O.H., Gerostamoulos D., Woodford N.W. (2019). Cannabis as a cause of death: a review. Forensic Sci. Int..

[2] Darke S., Duflou J., Farrell M., Peacock A., Lappin J. (2020). Characteristics and circumstances of synthetic cannabinoid-related death. Clin. Toxicol. Phila..

[3] Morrow P.L., Stables S., Kesha K., Tse R., Kappatos D., Pandey R. (2020). An outbreak of deaths associated with AMB-FUBINACA in Auckland NZ. EClinicalMedicine.

[4] Kleczkowska P., Smaga I., Filip M., Bujalska-Zadrozny M. (2016). Cannabinoid ligands and alcohol addiction: a promising therapeutic tool or a humbug?. Neurotox. Res..

[5] Langford A.M., Bolton J.R. (2018). Synthetic cannabinoids: variety is definitely not the spice of life. J. Forensic Leg. Med..

[6] Luethi D., Liechti M.E. (2020). Designer drugs: mechanism of action and adverse effects. Arch. Toxicol..

[7] Pava M.J., Woodward J.J. (2012). A review of the interactions between alcohol and the endocannabinoid system: implications for alcohol dependence and future directions for research. Alcohol.

[8] Basavarajappa B.S., Joshi V., Shivakumar M., Subbanna S. (2019). Distinct functions of endogenous cannabinoid system in alcohol abuse disorders. Br. J. Pharmacol..

[9] Cerne K. (2020). Toxicological properties of Delta9-tetrahydrocannabinol and cannabidiol. Arh. Hig. Rada Toksikol..

[10] Haspula D., Clark M.A. (2020). Cannabinoid receptors: an update on cell signaling, pathophysiological roles and therapeutic opportunities in neurological, cardiovascular, and inflammatory diseases. Int. J. Mol. Sci..

[11] Shahbazi F., Grandi V., Banerjee A., Trant J.F. (2020). Cannabinoids and cannabinoid receptors: the story so far. iScience.

[12] Hiley C.R., Kaup S.S. (2007). GPR55 and the vascular receptors for cannabinoids. Br. J. Pharmacol..

[13] Hetz C., Zhang K., Kaufman R.J. (2020). Mechanisms, regulation and functions of the unfolded protein response. Nat. Rev. Mol. Cell Biol..

[14] Tabas I., Ron D. (2011). Integrating the mechanisms of apoptosis induced by endoplasmic reticulum stress. Nat. Cell Biol..

[15] Urano F., Wang X., Bertolotti A., Zhang Y., Chung P., Harding H.P. (2000). Coupling of stress in the ER to activation of JNK protein kinases by transmembrane protein kinase IRE1. Science.

[16] Ron D., Walter P. (2007). Signal integration in the endoplasmic reticulum unfolded protein response. Nat. Rev. Mol. Cell Biol..

[17] Nakagawa T., Zhu H., Morishima N., Li E., Xu J., Yankner B.A. (2000). Caspase-12 mediates endoplasmic-reticulum-specific apoptosis and cytotoxicity by amyloid-beta. Nature.

[18] Garcia de la Cadena S., Massieu L. (2016). Caspases and their role in inflammation and ischemic neuronal death. Focus on caspase-12. Apoptosis.

[19] Carracedo A., Gironella M., Lorente M., Garcia S., Guzman M., Velasco G. (2006). Cannabinoids induce apoptosis of pancreatic tumor cells via endoplasmic reticulum stress-related genes. Cancer Res..

[20] Salazar M., Carracedo A., Salanueva I.J., Hernandez-Tiedra S., Egia A., Lorente M. (2009). TRB3 links ER stress to autophagy in cannabinoid anti-tumoral action. Autophagy.

[21] Salazar M., Carracedo A., Salanueva I.J., Hernandez-Tiedra S., Lorente M., Egia A. (2009). Cannabinoid action induces autophagy-mediated cell death through stimulation of ER stress in human glioma cells. J. Clin. Invest..

[22] Recouvreux M.V., Commisso C. (2017). Macropinocytosis: a metabolic adaptation to nutrient stress in cancer. Front Endocrinol (Lausanne).

[23] Maltese W.A., Overmeyer J.H. (2014). Methuosis: nonapoptotic cell death associated with vacuolization of macropinosome and endosome compartments. Am. J. Pathol..

[24] Commisso C., Davidson S.M., Soydaner-Azeloglu R.G., Parker S.J., Kamphorst J.J., Hackett S. (2013). Macropinocytosis of protein is an amino acid supply route in Ras-transformed cells. Nature.

[25] Moser T.S., Jones R.G., Thompson C.B., Coyne C.B., Cherry S. (2010). A kinome RNAi screen identified AMPK as promoting poxvirus entry through the control of actin dynamics. PLoS Pathog..

[26] Kim S.M., Nguyen T.T., Ravi A., Kubiniok P., Finicle B.T., Jayashankar V. (2018). PTEN deficiency and AMPK activation promote nutrient scavenging and anabolism in prostate Cancer cells. Cancer Discov..

[27] Yuan H.-X., Xiong Y., Guan K.-L. (2013). Nutrient sensing, metabolism, and cell growth control. Mol. Cell.

[28] Claycomb W.C., Lanson N.A., Stallworth B.S., Egeland D.B., Delcarpio J.B., Bahinski A. (1998). HL-1 cells: a cardiac muscle cell line that contracts and retains phenotypic characteristics of the adult cardiomyocyte. Proc. Natl. Acad. Sci. U. S. A..

[29] Abe E., Okawa S., Sugawara M., Watanabe S., Toyoshima I. (2007). Identification of ER membrane targeting signal of kinectin. Neurosci. Lett..

[30] Kano F., Kondo H., Yamamoto A., Kaneko Y., Uchiyama K., Hosokawa N. (2005). NSF/SNAPs and p97/p47/VCIP135 are sequentially required for cell cycle-dependent reformation of the ER network. Genes Cells.

[31] Nara A., Aki T., Funakoshi T., Uemura K. (2010). Methamphetamine induces macropinocytosis in differentiated SH-SY5Y human neuroblastoma cells. Brain Res..

[32] Lojpur T., Easton Z., Raez-Villanueva S., Laviolette S., Holloway A.C., Hardy D.B. (2019). Delta9-Tetrahydrocannabinol leads to endoplasmic reticulum stress and mitochondrial dysfunction in human BeWo trophoblasts. Reprod. Toxicol..

[33] Noritake K., Aki T., Funakoshi T., Unuma K., Uemura K. (2015). Direct exposure to ethanol disrupts junctional cell-Cell contact and Hippo-YAP signaling in HL-1 murine atrial cardiomyocytes. PLoS One.

[34] Rani S., Sreenivasaiah P.K., Kim J.O., Lee M.Y., Kang W.S., Kim Y.S. (2017). Tauroursodeoxycholic acid (TUDCA) attenuates pressure overload-induced cardiac remodeling by reducing endoplasmic reticulum stress. PLoS One.

[35] Gomes M.D., Lecker S.H., Jagoe R.T., Navon A., Goldberg A.L. (2001). Atrogin-1, a muscle-specific F-box protein highly expressed during muscle atrophy. Proc. Natl. Acad. Sci. U. S. A..

[36] Muller C., Morales P., Reggio P.H. (2018). Cannabinoid ligands targeting TRP channels. Front. Mol. Neurosci..

[37] O’Sullivan S.E. (2016). An update on PPAR activation by cannabinoids. Br. J. Pharmacol..

[38] Martin B.R. (1986). Cellular effects of cannabinoids. Pharmacol. Rev..

[39] Makriyannis A., Yang D.P., Griffin R.G., Das Gupta S.K. (1990). The perturbation of model membranes by (-)-delta 9-tetrahydrocannabinol. Studies using solid-state 2H- and 13C-NMR. Biochim. Biophys. Acta.

[40] Jayashankar V., Edinger A.L. (2020). Macropinocytosis confers resistance to therapies targeting cancer anabolism. Nat. Commun..

[41] Husain S. (1989). Effects of delta-9-tetrahydrocannabinol on in vitro energy substrate metabolism in mouse and rat testis. Physiol. Behav..

[42] Dando I., Donadelli M., Costanzo C., Dalla Pozza E., D’Alessandro A., Zolla L. (2013). Cannabinoids inhibit energetic metabolism and induce AMPK-dependent autophagy in pancreatic cancer cells. Cell Death Dis..

[43] Florey O., Overholtzer M. (2019). Macropinocytosis and autophagy crosstalk in nutrient scavenging. Philos. Trans. R. Soc. Lond. B, Biol. Sci..

[44] Salazar M., Lorente M., Garcia-Taboada E., Hernandez-Tiedra S., Davila D., Francis S.E. (2013). The pseudokinase tribbles homologue-3 plays a crucial role in cannabinoid anticancer action. Biochim. Biophys. Acta.

[45] Costa L., Amaral C., Teixeira N., Correia-da-Silva G., Fonseca B.M. (2016). Cannabinoid-induced autophagy: protective or death role?. Prostaglandins Other Lipid Mediat..

[46] Sarkar Bhattacharya S., Thirusangu P., Jin L., Roy D., Jung D., Xiao Y. (2019). PFKFB3 inhibition reprograms malignant pleural mesothelioma to nutrient stress-induced macropinocytosis and ER stress as independent binary adaptive responses. Cell Death Dis..

