# Supplementary material for: Possible roles of AMPK and macropinocytosis in the defense responses against Δ9-THC toxicity on HL-1 cardiomyocytes
Source: Toxicol Rep. 2021 Apr 30;8:980–7. doi: 10.1016/j.toxrep.2021.04.014 (PMC8131391; doi:10.1016/j.toxrep.2021.04.014)
Supplement: Supplementary file 1 [file mmc1.docx]

**S1 Table**

Antibodies used for western blot analysis.

| Antibody | Animal and type | Dilution | Source and identifier |
| --- | --- | --- | --- |
| Anti-BIP | Rabbit monoclonal | 1:1000 | Cell Signaling Technology, #3177 |
| Anti-ATF4 | Rabbit monoclonal | 1:1000 | Proteintech, 10835-1-AP |
| Anti-ATF6 | Rabbit monoclonal | 1:1000 | Proteintech, 24169-1-AP |
| Anti-CHOP | Rabbit monoclonal | 1:1000 | Cell Signaling Technology, #2895 |
| Anti-cleaved-caspase3 | Rabbit monoclonal | 1:1000 | Cell Signaling Technology, #9661 |
| Anti-cleaved-caspase12 | Rabbit monoclonal | 1:1000 | Cell Signaling Technology, #2202 |
| Anti-AMPKα | Rabbit monoclonal | 1:1000 | Cell Signaling Technology, #5831 |
| Anti-phospho-AMPKα | Rabbit monoclonal | 1:1000 | Cell Signaling Technology, #2535 |
| Anti-actin | Rabbit monoclonal | 1:1000 | Sigma, #A2006 |

**S2 Table**

Primers used for quantitative real-time PCR.

| Gene | Forward | Reverse |
| --- | --- | --- |
| BIP | GTGGTGAGACCAGAACCGT | ACAGTGAACTTCATCATGCCG |
| ATF4 | CTCATGGGGCCTTTAGGACG | GTGGTCACGTGATCCTACCG |
| ATF6 | TGGAGTCGCCTTTTAGTCCG | GCTGCATCAAAGTGCACATCA |
| CHOP | CCTGAGGAGAGAGTGTTCCAG | GACACCGTCTCCAAGGTGAA |
| MYH-6 | GCAGGCCCTGGCTCTTCAGC | TCCCGGGACAGGTCAGAGCG |
| MYH-7 | CCTGCTGTTTCCTTACTTGCTACCC | CTTCCGCAGGAAGGGG |
| Atrogin1 | GCAAGTCTGTGCTGGTGGGCA | CAGGTCCCGCCCGTCACTCA |
| GAPDH | GTGCAGTGCCAGCCTCGTCC | GCCACTGCAAATGGCAGCCC |
